# Sherwood’s New Molar Forceps

**Published:** 1862-12

**Authors:** 


### SHERWOOD'S NEW MOLAR FORCEPS.

HAVE you seen them? No! Indeed sir, you can't do without them! and your patients can't; and the neighborhood in which you live can never afford to be so far behind the times as to do without them. Send right along and get them. They are the *gospel* according to Sherwood—that is, they are the symbols of *good tidings* to the afflicted. Get them direct from Sherwood. "None others genuine." All imitations of them are *apochryphal*, etc., etc., *ad lib.*

Now reader, do you think Mr. Sherwood pays me for this notice? Certainly he does—pays in advance, too. Why, he invented the forceps and *sold* them to me at two, or three or four dollars apiece; and as they are worth at least twenty-five, and as after months of experience with them I would give that rather than do without them, of course I am more than paid. That's the only pay, good reader—the only kind that can call forth these "humble effusions." W.

